# Women's Safety and Gender-Based Violence in the Republic of North Macedonia

**DOI:** 10.3389/fpubh.2020.00033

**Published:** 2020-02-21

**Authors:** Fimka Tozija

**Affiliations:** ^1^Institute of Public Health of Republic of North Macedonia, Saints Cyril and Methodius University of Skopje, Skopje, North Macedonia; ^2^Faculty of Medicine, Saints Cyril and Methodius University of Skopje, Skopje, North Macedonia

**Keywords:** women, safety, violence, policy, prevention

## Abstract

Violence against women in the Republic of North Macedonia is the most common form of human rights violation, and women's safety is thus a high-priority public health problem. There have been significant achievements in the area of policy development: legislation harmonization for human rights protection, prevention of violence against women, protocols for the treatment and support of female victims, especially those with disabilities, and further collaboration and coordination between different sectors. In practice, there is still a need to establish a system of institutions for effective prevention, protection, gathering of evidence, and support of women victims of gender-based violence in addition to the prosecution of perpetrators. Policies for the improvement of women's safety should be considered as a priority and undertaken at an individual, relationship, community, and society level.

## Introduction

Women's safety is a global public health problem (1), and “Global estimates published by World Health Organization (WHO) indicate that about 1 in 3 (35%) of women worldwide have experienced either physical and/or sexual intimate partner violence or non-partner sexual violence in their lifetime. Worldwide, almost one third (30%) of women who have been in a relationship report that they have experienced some form of physical and/or sexual violence by their intimate partner in their lifetime. Globally, as many as 38% of murders of women are committed by a male intimate partner” (2). However, some national “studies show that up to 70% of women have experienced physical and/or sexual violence from an intimate partner in their lifetime” (3).

Violence has a negative effect on women's physical, mental, sexual, and reproductive health (1). Evidence shows that women who have experienced physical or sexually intimate partner violence report higher rates of depression, abortion, and acquiring HIV compared to women who have not (3).

Violence against women in the Republic of North Macedonia (further in the text Macedonia) is the most common form of human rights violation against women, and it has a negative impact on health, causing injuries, death, as well as psychological trauma (4). Women's safety is thus a severe public health problem of high priority (5–7).

Public health and human rights approaches have been applied to analyze the magnitude, burden, and risk factors of violence against women in Macedonia and to give actionable recommendations for policy interventions for the purpose of prevention. The public health approach is a science-based, multi-disciplinary approach for understanding and preventing violence, with coordinated action to be undertaken by different sectors (1).

## Magnitude, Burden, and Risk Factors for Violence Against Women

In Macedonia, due to inappropriate evidence of violence and a lack of systematic follow-up of the victims of violence, within health, social, educational, and other institutions in the country, it is not possible to give an objective assessment of the burden of violence against women (8).

The official data from the police showed that, in 2004, there were 2,434 complaints of domestic violence of which 1,000 victims were wives, 460 parents, and 175 were children and other family members (aunts, uncles, and husbands) in big families with members spanning several generations. In most of the cases, violence was linked to alcohol abuse. Centers for social work have provided services to 409 victims of family violence in 2004 and 2005, and 85 victims of violence were accommodated in the four shelter centers in Skopje, Kochani, Bitola, and Strumica (8).

A survey on all forms of domestic violence that was conducted by the non-governmental Organization for the Emancipation, Solidarity, and Equality of Women on a sample of 850 rural and urban women (1.3%0 of the total female population in Macedonia) in 2000 showed that 61.5% of the interviewed women reported psychological violence, 23.9% physical violence, and 5% sexual violence (9). Findings from the second survey conducted by the same organization in 2006 were similar; it surveyed a sample of 1,573 households, of which 1,432 were women, with dominant psychological violence, reported by 56.4% of the respondents, followed by physical violence (17.7%), while 10.6% of those interviewed reported sexual violence. In 2006, reported sexual violence was increased, with one report of sexual violence for every 1.7 reports of physical violence, which is significantly higher compared to the 2000 survey, which had one report of sexual violence for every 4.6 reports of physical violence (10).

An intimate partner was identified by women as the most frequent perpetrator in 43.9% of reported violence against women in the community injury survey conducted on a sample of 1,000 households in Macedonia in 2008 (11).

### Characteristic of Violence by Age

The Global School-Based Student Health Survey has shown that “overall 10.0% of students aged 13–15 years had been bullied on 1 and more days during the past 30 days, while 8.6% of students seriously considered attempting suicide during the past 12 months; male students (6.8%) were less likely than female students (10.5%) to seriously consider attempting suicide” (12).

Women at the age of 25–40 are most exposed to violence, and male perpetrators are between 40 and 44 years old (8). The prevalence of domestic violence against women is 39.4% with higher rates among younger women and the highest rates being among adolescents between 15 and 18 years of age (13).

### Violence by Ethnicity

Ethnic differences in violent behavior seem to exist: Roma women reported being mostly exposed to physical violence, while Albanian women reported being exposed to psychological violence (8).

Violence against women is more frequent in women with lower education among Macedonian and Serbian women. Physical violence has the highest prevalence rate among Roma women, while sexual violence is most prevalent among Turkish women (13).

The prevalence rate of domestic violence against women is higher in other South East European countries, as has been shown in several studies. In Albania, the prevalence of domestic violence against women (during life) is 59.4%, whereby psychological violence is most common (58.2%) followed by physical violence (23.7). Almost every second woman in Bosnia and Herzegovina (47.2%) has experienced at least one form of violence since age of 15, mostly psychological (41.9%) and physical (24.3). Data obtained from a study conducted in Serbia in 2010 show that the overall prevalence of violence against women is 54.2%, with psychological violence at 48.7% and physical violence at 21.6% (14).

The official number of violent deaths in females in Macedonia has decreased; homicides have gone down from 12 in 2007 to 9 in 2017 and suicides from 45 in 2007 to 32 in 2017, and this is almost three times lower than in males (15). Twenty-eight cases of femicides were registered within between 2001 and August 2016, and in 60% of cases the femicide was carried out by the woman's intimate partner and in 80% it occurred in their home (16).

Evidence suggests that certain characteristics in women, such as sexual orientation, disability status, or ethnicity, and some contextual factors, such as humanitarian crises, including conflict and post-conflict situations, may increase women's vulnerability to violence (3) and may also lead to new forms of violence against women (2).

### Violence by Perpetrators

The main risk factors for violence against women in Macedonia, as stated by the perpetrators, are poor economic status of perpetrator, low education, unemployment, exposure to domestic violence against their mothers, harmful use of alcohol (every fourth perpetrator), unequal gender norms, including attitudes accepting violence, and a sense of entitlement over women (8). The violent behavior of the perpetrator can be a risk factor for intimate partner violence as a function of disposition and situation (and their interaction) (17).

The main risk factors for victims of intimate partner violence as reported by the women are low levels of education, lack of employment for the woman, exposure to mothers being abused by a partner, experienced child abuse, and attitudes accepting violence, male privilege, and women's subordinate status (8, 18).

## Policy Implications—the State Response

The State response in addressing the problem of violence against women was in line with the resolution WHA67.15 from May 2014 (19) and the Global plan of action to strengthen the role of the health system in addressing interpersonal violence, in particular against women and girls as well as against children in general (20), and the United Nations' Sustainable Development Goals (SDGs) and related targets as per Agenda 2030 (21) through policy development; this has included harmonization of legislation for the prevention of violence against women, protocols for treatment and support of female victims, and collaboration and coordination of different sectors, policy development, and capacity building.

The Government has been undertaking evidence-based policy interventions that are in line with WHO key recommendations (19, 22) at a national level to achieve the SDG targets related to violence (20, 21) by strengthening data collection and surveillance in relation to violence against women; developing and implementing comprehensive and evidence-based national strategies and action plans; integrating violence prevention policies from different sectors; strengthening multisectoral coordination; and collaboration to provide comprehensive services for victims based on evidence; enforcing existing laws and reviewing their quality; and building on their capacity for the prevention of violence against women.

## Legislation Harmonization and Enforcement

The Republic of North Macedonia is a signatory of the international acts from the United Nations (UN), the Council of Europe (CoE), and the European Union (EU) for the protection of human rights and prevention of discrimination, torture, and maltreatment, which are incorporated in the national legislation (23). The most important section of the series of adopted UN documents are the Universal Declaration for Human Rights (24); the Covenant on Civil and Political Rights and its Protocols (ratified in 1994) (25); the Covenant on Economic, Social and Cultural Rights (ratified in 1994) (26); the Convention against Torture (ratified in 1997) (27); International Convention for the Elimination of all Forms of Racial Discrimination (28); the Convention on the Elimination of all Forms of Discrimination against Women (CEDAW) (29); the Convention on the Rights of the Child and its Protocols (ratified in 1993) (30); the Convention relating to the Status of Refugees (31); Convention on the Rights of Persons with Disabilities (CRPD) (32); Declaration on the Rights of the Patient (33); Charter on the Right to Health (34); International Convention on the Protection of the Rights of all Migrant Workers and Members of Their Families (35) as well as by the Council of Europe's Convention for the Prevention of Torture and Inhuman or Degrading Treatment or Punishment (36), and the European Convention on Human Rights and its Five Protocols (ratified in 1997) (37). One of the most important documents of CoE that specifically refer to violence against women and domestic violence is the Istanbul Convention signed in 2011 and ratified in 2018 (38).

The existing national legislative provisions are, to a great extent, in compliance with the principle of gender equality and the international instruments on the protection of women's human rights. Macedonia ratified the CEDAW Convention on January 18th, 1994, and the Optional Protocol to the same Convention and made no reservations (39).

The Constitution (40) stipulates that fundamental values of the constitutional order include “rights and freedoms of a citizen recognized by international law and determined by the Constitution,” and states that “Citizens of the Republic of Macedonia shall be equal in their freedoms and rights, regardless of their gender, race, color, national and social origins, political and religious beliefs, property and social status.”

Citizens shall be equal in the Constitution and the laws: Criminal Code (41); Law on Labor Relations (42); Family law (43); Law on Social Protection (44); Health Care Law (45); Law on Patient Rights Protection (46); and the Law on Equal opportunities for Women and Men (47). There is no general definition of discrimination and of a gender discrimination in the national legislation.

In 2003 and 2004, the State made amendments on the actual Family Law (43) and Law for Social Protection (44) as well as on the Crime Code (41) for secondary prevention, particularly sanctioning the perpetrators and protection of victims of domestic violence (18). A new law for prevention and protection from domestic violence was adopted in 2014 (48) and bylaws for the implementation of this law for relevant sectors were developed in 2015. The new Law for Evidence in Health (49) was adopted in 2009, and this means that the reporting of violence has become mandatory for health professionals in a special individual report for violence.

The Annual Report of the Helsinki Committee for 2012 shows that there were serious violations to the freedoms and rights of citizens as well as drastic violations to the principle of rule of law and the legal state; this was not only in the form of systemic problems in the functioning of the bodies and institutions but also in the form of the abuse of their competencies (50).

## Policy Development and Interventions

Violence prevention was set as priority in the collaboration between the Ministry of Health and the World Health Organization (WHO) since 2004 in Biennial collaborative agreements. The WHO National focal point for violence and injury control and prevention was appointed by the Minister of Health in 2003, thus starting the collaboration with the WHO organizing the national conference against violence against women on November 25th, 2003.

The Department for Violence and Injury Control and Prevention was established within the Institute of Public Health in 2004 as a lead agency for violence prevention in the health sector, and it was later in 2012 inaugurated as the Safe Community Affiliate Support Center.

The first shelter center was opened in 2004 in Skopje for the protection of victims of domestic violence. The process was followed by the opening of five more centers in the country and a national SOS line financed by the Government. Training of social workers and health professionals was also conducted (8).

In the institutional framework, the sector for equal opportunities was established within the Ministry of Labor and Social Policy for the improvement of the status of women. There is also the national commission on equal opportunities for women and men, which was established in 2006 as a regular parliamentary commission (39).

The Governmental National Coordination Body for Domestic Violence Prevention was established with Government approval in 2009, and it includes representatives from the Ministries of Health, Labor and Social Policy, the Interior, Education, Justice, the Institute of Public Health, as well as other non-governmental organizations, WHO and other stakeholders responsible for the implementation of the National Strategy for protection against domestic violence 2008–2011 (51).

The Joint UN Program “Strengthening National Capacities to Prevent Domestic Violence” started in December 2008 and finished in August 2012, and it provided technical and financial assistance to the National Coordination Body in the implementation of the National Strategy for protection from domestic violence. UN agencies UNDP, UNFPA, WHO, UNICEF, and UNIFEM have worked together with the national partners: Ministries of Labor and Social Policy, the Interior, Justice, Health, Education, and Science, providing funding of 2,458,000 US dollars from the Government of the Netherlands, 958,000 US dollars from the UN Trust Fund, and a further 43,000 US dollars from UN agencies. A total of 70,000 US dollars in kind have also been provided by Government of the Republic of Macedonia (52).

Specific national policies for domestic violence prevention have been developed to address violence against women, and annual action plans have also been prepared for implementation: a National Strategy for Protection Against Domestic Violence 2008–2011 (51) was implemented and coordinated by the National Coordination Body, and a second National Strategy for Protection Against Domestic Violence 2012–2015 (53) has also been developed and implemented.

Protocols against child abuse and neglect, sexual abuse, and pedophilia have been prepared as well (54). The Joint Protocol for treatment in case of domestic violence was prepared in collaboration with the Ministries of Health, Labor and Social Welfare, the Interior, Education, and Justice, as well as NGOs, UNDP, UNFPA, WHO, UNIFEM, and other UN agencies in 2010 (55). A protocol for the intersectoral collaboration of responsible institutions and associations for protection against domestic violence was developed in 2015 for the implementation of the new legislation, and it stipulates the cooperation between police, health organizations, centers for social work, schools, and NGOs in the frame of the protection of female victims of domestic violence (56). A Counseling center for perpetrators was established in Clinic for psychiatry in 2012.

In the context of the recent ratification of the European Council Istanbul Convention, the Government opened three multi-sectoral sexual assault referral centers in the hospitals in Tetovo, Kumanovo, and the University Obstetrics and Gynecology Clinic in Skopje for psychosocial and legal support as well as for the safety and security of the victims of sexual violence, applying a standard operating procedure. In the next 5 years, the Government will open seven more centers (57).

The First Family Center established in 2013 in Skopje as a specialized counseling center for support and prevention against domestic violence is an example of good cooperation between the civil sector, local government, and business sector, providing free, confidential, and high-quality counseling and psychotherapy services for victims and perpetrators of domestic violence (58).

## Capacity Building for Violence Prevention

The WHO Training Education Advancing Collaboration in Health Violence and Injury Prevention modular training curriculum on violence prevention and control has been completed by 2,390 health professionals and 60 university professors within the period 2009–2012. Training was financially supported by WHO within the UN Joint Program mentioned above. Two hundred twenty doctors were trained in 1 day workshops for evidence of violence and web register in Skopje in 2012 (59).

Guidelines for referral mechanisms for professionals were prepared in 2010 and 2-day capacity-building workshops for the implementation of the Joint Protocol for domestic violence prevention were organized in eight municipalities in the period 2011–2012, and these were supported by the UN Joint Program. A total of 200 professionals from different sectors were trained: health workers, social workers, policemen, teachers, municipal staff, etc.

Three 1-day capacity-building workshops for the protocol for intersectoral collaboration for domestic violence prevention were organized in 2015, and 90 doctors, nurses, social workers, and other workers were trained.

In 2016, the Ministry of Health, in cooperation with NGO HERA, UNFPA, the Center for Family Medicine, and the Institute for Public Health, accredited a training package for gender-based violence with nine modules for 2-day training for health professionals. Over 200 medical professionals have completed this training, while 40 primary health care physicians completed a 2-day training program for gender-based violence in persons with disabilities in 2019.

## Actionable Recommendations

Despite the progress made in violence control and prevention and safety promotion, further efforts and a more strategic approach are needed in the years ahead (22), especially for addressing violence against women and achieving the SDG targets related to violence.

The future key challenges in the prevention of violence against women in Macedonia in the near future will be to implement the legislation for the prevention of domestic violence as well as the recommendations of the WHO World Report on Violence and Health (1) through the various activities: developing and implementing evidence-based interventions for safety promotion and reducing socioeconomic inequalities; addressing violence and achieving SGDs; improving data collection and research for violence among women and developing an integral information system for violence surveillance; strengthening capacity and trainings for professionals for prevention of violence against women at all levels focusing on primary prevention; and promoting gender and social equality and empowerment.

Advocacy and empowerment counseling interventions, education, and home visitation are promising in the prevention or reduction of intimate partner violence against women (2).

A multisectoral integrated approach is required for gender-based violence reduction at both the legislative and community level, dealing with common risk factors as well as the cultural and societal influences behind perpetrator behavior and victim response.

Overall strengthening of the main functions, and especially the development of violence-prevention policies and legislation and establishing strong monitoring, will add significant value to the integrated system for the prevention of violence against women while including health in all policies.

## Conclusion

There are achievements in policy development for the prevention of violence against women as well as capacity-building and multisectoral collaboration between governmental, civil society, and international organizations with legislative and institutional changes. But there is still a need for the Government to provide resources and undertake measures and activities to respond to cultural change in social systems and to improve efficiency and effectiveness of legal mechanisms for the protection of women. Policies for the improvement of women's safety should be considered as a priority and should be undertaken at the individual, relationship, community, and society level.

## Author Contributions

The author confirms being the sole contributor of this work and has approved it for publication.

### Conflict of Interest

The author declares that the research was conducted in the absence of any commercial or financial relationships that could be construed as a potential conflict of interest.
